# Human Papillomavirus Vaccination in Adult Survivors of Childhood, Adolescent, and Young Adult Cancers: A Missed Opportunity

**DOI:** 10.7759/cureus.76177

**Published:** 2024-12-22

**Authors:** Chaitali S Dagli, Betelihem B Tobo, Mrudula Nair, Nada Al-Antary, Samantha H Tam, Nosayaba Osazuwa-Peters, Eric Adjei Boakye

**Affiliations:** 1 Epidemiology, Birmingham School of Medicine, University of Alabama, Birmingham, USA; 2 Community and Family Medicine, Howard University College of Medicine, Washington, USA; 3 Public Health Sciences, Henry Ford Health System, Detroit, USA; 4 Otolaryngology, Henry Ford Health System, Detroit, USA; 5 Otolaryngology, Duke University School of Medicine, Durham, USA; 6 Otolaryngology and Public Health Sciences, Henry Ford Health System, Detroit, USA

**Keywords:** adolescent and young adult (aya), cancer survivors, hpv vaccination, human papillomavirus, national health interview survey, vaccine coverage

## Abstract

Introduction

Studies assessing human papillomavirus (HPV) vaccination uptake in survivors of childhood, adolescent, and young adult (CAYA) cancers are sparse. We examined HPV vaccine uptake between survivors of CAYA cancer aged 18-35 and 18-35-year-old respondents without a cancer diagnosis in the United States.

Methods

We used the 2017-2018 National Health Interview Survey, a national, annual cross-sectional national dataset that monitors health-related information on the non-institutionalized civilian population in the United States. Outcome variables included: 1) self-reported initiation of the HPV vaccine, defined as having received ≥1 dose, and 2) self-reported completion of the HPV vaccine, defined as having received ≥3 doses. The exposure variable was cancer survivorship, dichotomized as CAYA cancer survivors (those diagnosed with cancer during childhood, adolescence, or young adulthood) versus non-cancer survivors (no cancer diagnosis). -Weighted multivariable logistic regression models estimated the association between cancer survivorship and HPV vaccine initiation and completion, adjusting for socioeconomic covariates and factors related to healthcare access.

Results

A total of 2677 respondents were included in the study, of which 177 (5.3%) were CAYA cancer survivors. Overall, 28.0% of the study cohort initiated and 17.1% completed the HPV vaccine series. When stratified by cancer survivorship, initiation of the HPV vaccine (27.1%) and completion of the vaccine series (20.3%) among CAYA cancer survivors were comparable to respondents without cancer diagnosis (initiation: 28.1%, completion: 16.9%). After we controlled for covariates, cancer survivorship had neither a significant association with initiation of HPV vaccine (aOR=1.12; 95% CI, 0.71-1.79; P=0.6242) nor completion of HPV vaccine (aOR=1.37; 95% CI, 0.84-2.22; P=0.2055).

Conclusions

There was low HPV vaccination initiation and completion among both cohorts. CAYA may benefit the most from HPV vaccination, given that they are at a higher risk of developing secondary HPV-related cancer.

## Introduction

Each year, approximately 48000 cases of human papillomavirus (HPV)-associated cancers, including cervical, anal, oral, penile, vaginal, and vulvar, are attributed to HPV in the United States [1]. Approximately 91% of cervical and anal cancers are attributable to HPV, 75% of vaginal and 70% of oropharyngeal cancers are attributable to HPV, and 69% of vulvar and 63% of penile cancers are attributable to HPV [1]. Persistent HPV infections are also associated with an increased risk of developing second primary cancers [2-4]. Routine HPV vaccination is recommended for adolescents between 11 and 12 years old, although it can be given to children as young as nine years old [5]. Additionally, it is recommended that routine catch-up vaccination be administered for individuals aged 13-26 years. The recommended model for those who fall in the category of 27-45 years of age is shared clinical decision-making regarding vaccination [5]. HPV vaccination has the potential to prevent over 90% of cancers associated with HPV [6]. However, HPV vaccination among survivors of childhood, adolescent, and young adult (CAYA) cancers is suboptimal [7].

There were an estimated 678000 survivors of childhood and adolescent cancers in the United States in 2019 [8]. Previous studies demonstrated that survivors of CAYA cancers remain at higher risk of developing second primary cancers over their lifetime [9,10]. Given CAYA cancer survivors are a growing part of society, they will benefit from increased HPV vaccination. Examining HPV vaccine uptake among survivors of CAYA cancers will inform evidence-based interventions to improve this group’s low vaccination rates. However, studies assessing HPV vaccination coverage in survivors of CAYA cancers are lacking. We assessed HPV vaccine initiation and completion among survivors of CAYA cancers compared to respondents without a cancer diagnosis in the United States.

## Materials and methods

Data source and study sample

We acquired data from the 2017-2018 National Health Interview Survey (NHIS), a nationally representative sample of the civilian, non-institutionalized United States population conducted by the National Center for Health Statistics. The NHIS uses a multistage clustered sample design, which oversamples Asian, Black, and Hispanic populations. The data were collected through a personal household interview. One civilian adult per family is randomly selected for a detailed interview, which includes health status, health behavior, and healthcare utilization. Details of survey development, design, and methodology have been published elsewhere [11]. Data are publicly available and approved by the National Center for Health Statistics, and all respondents provided verbal consent. Because the study used deidentified and publicly available data, Institutional Review Board approval was exempt. We included individuals aged 18 to 35 years. Our upper age limit was 35 years; this subgroup of adults would have achieved eligibility for HPV vaccination after its approval in 2006 in the United States. A total of 177 survivors of CAYA cancer aged 18-35 were selected, and 2500 respondents of the same age group without a cancer diagnosis were selected randomly. This resulted in a sample size of 2677 being used in the study analysis.

Measures

The outcome variables were HPV vaccine initiation and completion. HPV vaccine initiation was assessed with the question “Ever received HPV shot/vaccine?” Respondent responses were dichotomized (yes or no). Respondents who responded yes were subsequently asked how many doses of the HPV vaccine they had received. HPV vaccine initiation was defined as receipt of ≥1 dose and completion as receipt of ≥3 doses of the vaccine. The independent variable was cancer survivorship classified as survivors of CAYA cancer (18-35 year-old respondents diagnosed with cancer as a child, adolescent, or young adult (0-35 years old)) and non-cancer survivors (18-35 year-old respondents without a cancer diagnosis).

Covariates included age at the time of interview, sex (male or female), race/ethnicity (non-Hispanic white, non-Hispanic black, Hispanic, or non-Hispanic other), marital status (married or not married), education level (less than high school, high school graduate, some college, or college graduate), insurance status (yes or no), and number of health care visits in the past year (none, 1-5, or ≥6).

Statistical analysis

Descriptive statistics were used to analyze respondents’ characteristics. We compared respondents' characteristics by cancer survivorship using χ² tests. Weighted multivariable logistic regression models examined the association between cancer survivorship and HPV vaccine initiation and completion, controlling for sociodemographic and healthcare access-related factors (age at the time of interview, sex, race/ethnicity, marital status, education level, insurance status, and number of healthcare visits). All analyses were performed in SAS 9.4 (SAS Institute Inc., Cary, NC) using survey-specific procedures, which incorporate survey sampling weights to account for the complex sampling design used in the NHIS and to provide representative estimates of the United States population. The statistical significance level was defined as two-sided P<05.

## Results

A total of 2677 survey respondents were included in the study. Overall, the average age was 27 (0.10) years, there was a similar distribution of gender, most were non-Hispanic White (62.9%), and had health insurance (85.3%; Table 1). When stratified by cancer survivorship, survivors of CAYA cancer tended to be females, non-Hispanic Whites, married, or had a higher number of healthcare provider visits (Table 1).

**Table 1:** Study sample characteristics: 2017-2018 NHIS (n=2677) HPV: human papillomavirus; CAYA: childhood, adolescent, and young adult; NHIS: National Health Interview Survey

	Unweighted n (Weighted %)			P-values
	Overall	CAYA cancer survivors		
		Yes	No	
Age at the time of the interview, mean (SD)	27.42 (0.10)	29.42 (0.32)	27.28 (0.10)	<0.0001
Sex				<0.0001
Male	1257 (47.0)	57 (32.2)	1200 (48.0)	
Female	1420 (53.0)	120 (67.8)	1300 (52.0)	
Race/ethnicity				0.0003
Non-Hispanic White	1684 (62.9)	136 (76.8)	1548 (61.9)	
Non-Hispanic Black	319 (11.9)	14 (7.9)	305 (12.2)	
Hispanic	462 (17.3)	24 (13.6)	438 (17.5)	
Non-Hispanic Other	212 (7.9)	3 (1.7)	209 (8.4)	
Marital status				0.0059
Married	1142 (42.7)	93 (52.5)	1049 (42)	
Not Married	1535 (57.3)	84 (47.5)	1451 (58)	
Education level				0.0005
College graduate or more	905 (33.8)	45 (25.4)	860 (34.4)	
Some college	943 (35.2)	79 (44.6)	864 (34.6)	
High school graduate	602 (22.5)	29 (16.4)	573 (22.9)	
Less than high school	225 (8.4)	24 (13.6)	201 (8.1)	
Health insurance				0.1862
Yes	2284 (85.3)	145 (81.9)	2139 (85.6)	
No	393 (14.7)	32 (18.1)	361 (14.4)	
Number of healthcare provider visits				<0.001
None	624 (23.3)	22 (12.4)	602 (24.1)	
1-5	1525 (57.0)	82 (46.3)	1443 (57.7)	
≥6	528 (19.7)	73 (41.2)	455 (18.2)	

**Figure 1:**
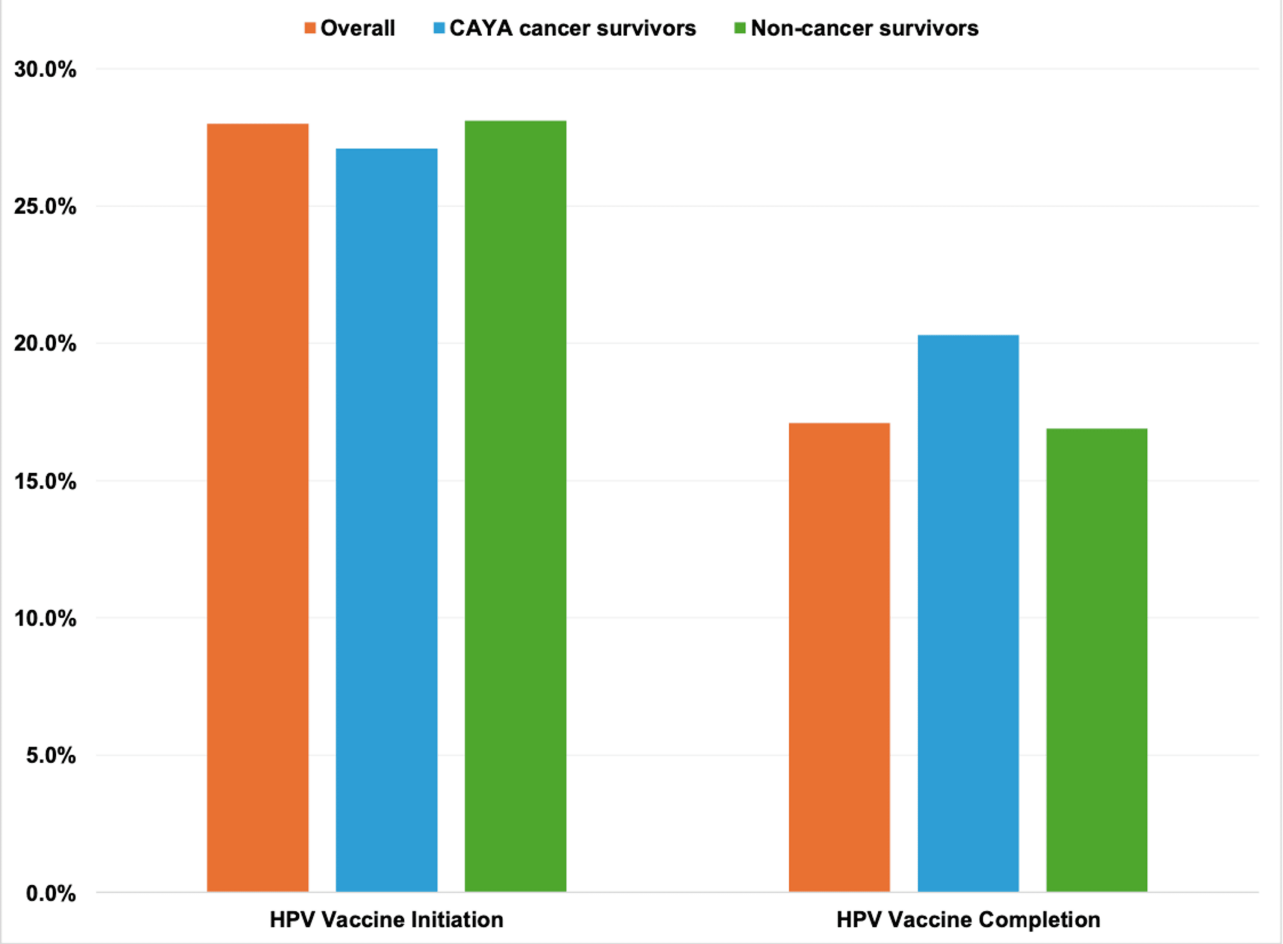
HPV vaccine initiation and completion, overall and by cancer survivorship: 2017-2018 NHIS (n=2677) HPV vaccine initiation among survivors of CAYA cancer versus non-cancer survivors (P=0.8081). HPV vaccine completion among survivors of CAYA cancer versus non-cancer survivors (P=0.3313). P-values were based on Chi-Square tests. NHIS: National Health Interview Survey; CAYA: childhood, adolescent, and young adult

Overall, the proportion of respondents initiating the HPV vaccine was 28.0% and completing the vaccine series was 17.1% (Figure 1). The proportion of respondents initiating the HPV vaccine who were survivors of CAYA cancer (27.1%) was comparable to that of non-cancer survivors (initiation: 28.1%, P=0.8081) (Figure 1). Similarly, the proportion of respondents completing the HPV vaccine series who were survivors of CAYA cancer (20.3%) was comparable to that of non-cancer survivors (completion: 16.9%, P=0.3313) (Figure 1).

**Figure 2:**
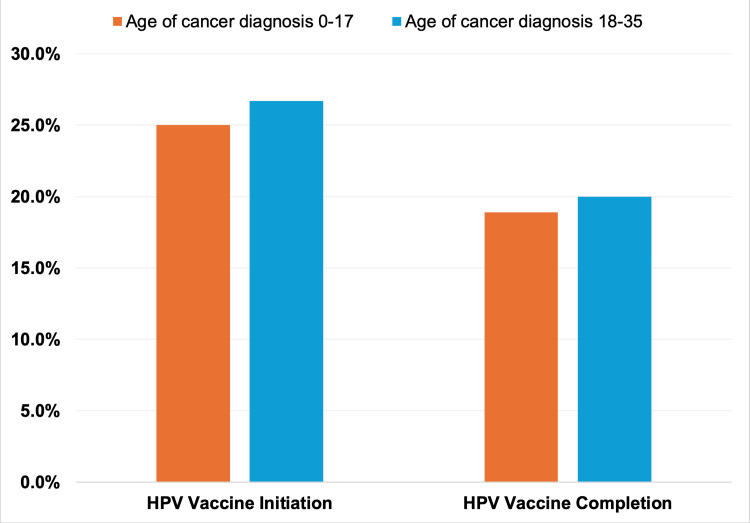
HPV vaccine initiation and completion by age of cancer diagnosis among CAYA survivors, 2017-2018 NHIS (n=177) HPV vaccine initiation among survivors of CAYA cancer diagnosed at ages 0-17 versus those diagnosed at ages 18-35 (P=0.8401). HPV vaccine completion among survivors of CAYA cancer diagnosed at ages 0-17 versus those diagnosed at ages 18-35 (P=0.8833). P-values were based on Chi-Square tests. NHIS: National Health Interview Survey; CAYA: childhood, adolescent, and young adult

In a sub-analysis assessing HPV vaccination among survivors of CAYA cancer based on the age of their cancer diagnosis, there were no significant differences in both initiation (P=0.8401) and completion (P=0.8833) between diagnosis ages of 0-17 and 18-35 years (Figure 2).

In the adjusted analyses, there were no significant differences in HPV vaccine initiation (aOR=1.12; 95% CI, 0.71-1.79; P=0.6242) or completion (aOR=1.37; 95% CI, 0.84-2.22; P=0.2055) between survivors of CAYA cancer and non-cancer survivors (Table 2). Older age was associated with increased odds of HPV vaccine initiation (aOR=1.17; 95% CI, 1.13-1.20) and completion (aOR=1.12; 95% CI, 1.08-1.16). Similarly, compared to respondents who had a college degree or higher, those with some college education (aOR_initiation_=1.53; 95% CI, 1.12-2.08; aOR_completion_=1.67; 95% CI, 1.19-2.36), high school diploma (aOR_initiation_=2.02; 95% CI, 1.40-2.90; aOR_completion_=1.99; 95% CI, 1.30-3.05), or less than high school graduate (aOR_initiation_=3.35; 95% CI, 1.88-5.97; aOR_completion_=3.59; 95% CI, 1.30-4.47) had higher odds of initiating and completing the HPV vaccine. Respondents who had not visited the healthcare provider over the past 12 months (aOR=2.32; 95% CI, 1.53-3.53) or visited the healthcare provider one to five times (aOR=1.47; 95% CI, 1.08-1.99) had higher odds of initiating the HPV vaccination compared to those who visited the healthcare provider ≥6 times.

**Table 2:** Factors associated with HPV vaccine initiation and completion, 2017-2018 NHIS (n=2677) HPV: human papillomavirus; CAYA: childhood, adolescent, and young adult; NHIS: National Health Interview Survey

	aOR (95% CI)	
	HPV vaccine initiation	HPV vaccine completion
CAYA cancer survivors		
Yes	1.12 (0.71, 1.79)	1.37 (0.84, 2.22)
No	Reference	Reference
Age at the time of the interview	1.17 (1.13, 1.20)	1.12 (1.08, 1.16)
Sex		
Male	Reference	Reference
Female	0.25 (0.19, 0.33)	0.26 (0.18, 0.36)
Race/ethnicity		
Non-Hispanic white	Reference	Reference
Non-Hispanic black	1.28 (0.87, 1.90)	1.40 (0.88, 2.24)
Hispanic	1.39 (0.96, 2.00)	1.31 (0.89, 1.93)
Non-Hispanic Other	1.31 (0.76, 2.28)	1.04 (0.56, 1.93)
Marital status		
Married	Reference	Reference
Not married	0.77 (0.59, 1.00)	0.90 (0.66, 1.21)
Education level		
College graduate or more	Reference	Reference
Some college	1.53 (1.12, 2.08)	1.67 (1.19, 2.36)
High school graduate	2.02 (1.40, 2.90)	1.99 (1.30, 3.05)
Less than high school	3.35 (1.88, 5.97)	3.59 (1.30, 4.47)
Health insurance		
Yes	0.98 (0.66, 1.45)	0.95 (0.62, 1.45)
No	Reference	Reference
Number of healthcare provider visits		
None	2.32 (1.53, 3.53)	1.53 (0.95, 2.44)
1-5	1.47 (1.08, 1.99)	1.13 (0.82, 1.58)
≥6	Reference	Reference

## Discussion

We found that vaccine uptake did not vary between survivors of CAYA cancer and non-cancer survivors and was very low for both groups. Notably, cancer survivorship did not significantly predict HPV vaccine series initiation and completion. Our study is consistent with others reporting this vulnerable population’s suboptimal HPV uptake [7,12]. Survivors of CAYA cancer might have potential barriers contributing to their suboptimal HPV vaccine uptake. These barriers could include patients’ skepticism regarding the efficacy of the vaccine, fear of its potential side effects, or fear of reliving past negative experiences by reestablishing contact with healthcare providers. Moreover, a lack of patients’ knowledge about their health could lead to the misperception that their immunocompromised state during their cancer journey continues longer than the research states. This misconception can hinder patients’ ability to accept the vaccine even after the recovery of their immune system. Another contributing factor is patients being labeled ineligible for vaccination during their cancer treatment, without any future follow-up to ensure vaccine uptake when eligible. As guidelines suggest vaccination to be held off for three to six months for those receiving immunosuppressive therapy, the conversation could be revisited when vaccination time is appropriate [13]. Furthermore, studies showed that during the early years following the completion of their cancer treatment, certain survivors of childhood cancer might lose follow-up with their primary care provider, potentially contributing to the low uptake of the HPV vaccine [14,15].

Low HPV vaccine uptake in survivors of CAYA cancer is a more serious public health concern given their heightened immunocompromised status and elevated risk for second primary cancers [9,10]. Enhanced communication between different specialties, primarily oncology, and primary care divisions, might be a key contributor to the timing of HPV vaccine initiation and completion among this age group. Primary care and oncology providers should encourage and recommend HPV vaccination and raise awareness of its crucial role among survivors of CAYA cancer and their families upon diagnosis. These providers should use every visit along the cancer care continuum as an opportunity to discuss and recommend the vaccine to eligible survivors of CAYA cancer and their children and family members if applicable. Additionally, with the expansion of vaccine eligibility up to 45 years, more oncology providers can be involved in patient education and vaccine recommendations. Moreover, primary care providers should use every clinical visit as an opportunity to discuss and recommend the HPV vaccine to eligible individuals without cancer diagnosis.

We found that individuals with lower levels of education attainment had lower odds of initiating and completing their HPV vaccine schedule compared to their higher-educated counterparts with college degrees or higher. This could be attributed to the increasing vaccine hesitancy occurring among individuals with higher socioeconomic backgrounds, which includes those who attain higher levels of education [16]. Second, providers may feel uncomfortable discussing clinical recommendations with someone who continually challenges those recommendations. Providers might not pursue patients beyond patients’ initial resistance or acceptance of their vaccine recommendations. Previous research showed that the frequency of HPV vaccine recommendations by providers is below the optimal national recommendations [17]. Furthermore, it was highlighted that the provider's perception of hesitancy could be discouraging them from routinely recommending the vaccine [18,19]. Thus, it is important to equip providers with the skills they need to navigate conversations with their patients who express vaccine hesitancy. Medical and continuing medical education programs should encompass providers’ access to skill-building in health communication with reluctant patients. Specifically, providers would benefit from cultivating skills in cultural humility and active listening skills, which would enable them to elucidate a patient’s unique barriers to vaccination and strategies to alleviate that patient’s concerns. Consistent discussion supported by evidence-based strategies with continuous follow-up could be used to address parental/patient hesitancy [20].

Our study found that respondents who had more frequent visits to their healthcare provider had lower odds of initiating their vaccine schedule, which is inconsistent with other findings [21]. Other studies have highlighted healthcare visits, specifically primary care visits, as major mechanisms for delivering multiple doses of the HPV vaccine [22,23]. In our sample population, it is possible that individuals with a higher number of healthcare visits were too ill for their providers to recommend HPV vaccination. Secondly, we had an adult population, meaning most of them were past the recommended age of 11-12 and, therefore, might not have received a provider recommendation for or been offered the vaccine. This means that a greater number of healthcare visits indicate patient interactions for reasons other than HPV vaccinations.

Limitations

There are limitations to our study. First, due to the small sample size, we were not able to conduct any sub-analyses by cancer type and sex. Second, due to the study’s cross-sectional study design, we are unable to make causal inferences. Third, there were variables we could not adjust for that could have affected the findings, such as provider recommendation for the vaccine, residential status (rural and urban), and religious beliefs. These variables were not collected in the NHIS dataset. Fourth, HPV vaccination data was self-reported, thus there is the possibility of recall and social desirability biases.

## Conclusions

In conclusion, among 18-35-year-olds in the NHIS dataset, there was low HPV vaccination initiation and completion among survivors of CAYA cancer and non-cancer survivors. Although survivors of CAYA cancer are at a higher risk of developing an HPV-related second cancer and may benefit the most from HPV vaccination, they were no more or less likely to initiate and complete the vaccine compared to their counterparts without a cancer diagnosis. During the immediate post-diagnosis and treatment initiation periods, vaccinations are often deferred until immunological recovery, underscoring the importance of revisiting this discussion once safety is ensured. The results of this study highlight the need for interventions that promote increased vaccination in this growing subpopulation and eligible individuals without a cancer diagnosis.
